# Orally Administered Edible Snail Extract Powder Enhances Skin Hydration via Hyaluronic Acid Synthesis and Barrier Gene Modulation in SKH‐1 Hairless Mice

**DOI:** 10.1002/fsn3.71087

**Published:** 2025-10-16

**Authors:** Chaerin Lee, Seoyoung Baek, Wonchul Lim, Tae‐Gyu Lim

**Affiliations:** ^1^ Department of Food Science & Biotechnology Sejong University Seoul Republic of Korea; ^2^ Department of Food Science & Biotechnology, and Carbohydrate Bioproduct Research Center Sejong University Seoul Republic of Korea

**Keywords:** edible snail extract powder, functional food, skin barrier, skin hydration, transepidermal water loss

## Abstract

SKH‐1 hairless mice were used as the in vivo model in this study to evaluate the efficacy of oral snail extract powder (SEP) on skin hydration and barrier enhancement. The skin serves as a primary barrier against external stimuli and plays a critical role in maintaining water homeostasis. Optimal skin hydration is essential for preserving barrier integrity and preventing dermatological conditions such as xerosis and atopic dermatitis. Although edible snail has demonstrated moisturizing effects when applied topically, its efficacy following oral administration has not been sufficiently characterized. In this study, we evaluated the moisturizing efficacy and safety of orally administered edible snail extract powder in SKH‐1 hairless mice. Animals were divided into a control group and three treatment groups receiving 20, 40, or 80 mg/kg B.W. of SEP daily for 13 weeks. Skin hydration and transepidermal water loss (TEWL) were measured periodically throughout the experiment. To investigate the underlying mechanisms, we analyzed the mRNA expression of genes related to hydration and barrier function, quantified skin hyaluronic acid (HA) levels via ELISA, and performed immunohistochemical staining. Oral SEP administration increased skin hydration and decreased TEWL, suggesting improved barrier function. Gene expression analyses revealed upregulation of Has1–3, Col1a1, Col3a1, Tgf‐β1, Flg, and Cers2, along with downregulation of Hyal1 and Acer1. These effects were supported by higher hyaluronic acid content and confirmed by altered protein expression of HAS2, HYAL1, and TGF‐β1. No significant changes were observed in body weight, food intake, or organ weights, and no adverse effects were observed at tested doses, supporting the safety of oral SEP administration. Overall, these findings suggest that orally administered SEP improves skin hydration and strengthens barrier integrity by regulating both molecular and tissue‐level pathways, highlighting its potential as a functional food ingredient for skin health.

## Graphical abstract

Immunohistochemical staining of dorsal skin tissues for HAS2, HYAL1, and TGF‐β1. Oral administration of SEP increased the expression of HAS2 and TGF‐β1 in a dose‐dependent manner, while reducing HYAL1 expression. These findings indicate that SEP promotes hyaluronic acid synthesis and extracellular matrix remodeling. The results support the moisturizing and barrier‐enhancing effects of SEP at the protein level.

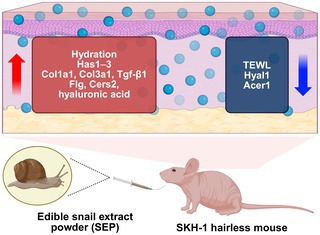

## 1 Introduction

The skin is the largest organ in the human body and serves various physiological defense functions, including blocking physical and chemical irritants from the external environment, preventing the invasion of pathogens, and minimizing water loss from the body (Bibi et al. 2025). Structurally, it consists of the epidermis, which is composed of multiple layers of keratinocytes, and the dermis, which contains blood vessels and connective tissues (Belkaid and Segre 2014).

The epidermis, the outermost layer of the skin, functions as a selectively permeable barrier and is composed of the stratum corneum (SC), stratum granulosum, and stratum basale (Baroni et al. 2012). It plays a crucial role in preventing the penetration of harmful substances from the external environment and regulating transepidermal water loss (TEWL). In contrast, the dermis consists of a complex supportive matrix that contributes to the skin's elasticity and water retention capacity, primarily composed of a structural network of polysaccharides and proteins (Seo et al. 2024; Shin et al. 2019). The ground substance of the dermis contains a lot of large molecules like glycosaminoglycans (GAGs) and proteoglycans, which are distributed among the elastic fibers. Among them, hyaluronic acid (HA) plays a key role in maintaining skin hydration due to its exceptional water‐binding ability (Papakonstantinou et al. 2012; Ruiz Martinez et al. 2020).

The SC, which is the outermost layer of the epidermis, is composed of corneocytes surrounded by lipids and denucleated keratinized cells. It plays a crucial role in moisture retention and protection against external irritants, as well as in maintaining the integrity of the skin barrier (Gorzelanny et al. 2006, 2020).

Under an electron microscope, abnormal thickening, cracking, and a disorganized structure characterize the compromised SC. This impairment of the skin barrier is a common pathological feature of various inflammatory skin diseases, including atopic dermatitis and psoriasis (Xie et al. 2024). One of the primary strategies for restoring skin barrier function is the use of moisturizers, which help adsorb and retain moisture not only on the skin surface but also within the deeper layers of the skin. Moisturizers are composed of occlusive, emollient, and humectant agents, which strengthen the skin's physical barrier by preventing water evaporation, softening the skin, and attracting moisture from the external environment, respectively (Rajkumar et al. 2023). Also, moisturizers help relieve xerosis, which is a condition where the skin becomes very dry and flakes because it doesn't have enough water in the outer layer (Draelos 2018).

Topical moisturizers are inherently constrained by the limited permeability of the SC, which impedes their capacity to reach and act upon deeper cutaneous layers. In contrast, oral supplementation affords systemic bioavailability, ease of administration, and the potential to modulate skin physiology via the gut–skin axis. These systemic effects provide distinct advantages over topical formulations in targeting multisite and deeper skin dynamics (De Almeida et al. 2023; Jimenez‐Sanchez et al. 2025).

Snails are terrestrial mollusks classified under the class Gastropoda, subclass Pulmonata, and phylum Mollusca. Snail mucus is a biologically active secretion with viscous and elastic properties, exhibiting strong adhesive and lubricating capabilities that allow it to adhere to various surfaces. Snail mucus has been used for medicinal purposes such as wound healing since ancient times, including in ancient Greece and during the Later Han dynasty in China. In recent years, it has garnered considerable attention due to its potential biological activities, including antioxidant, anticancer, and anti‐aging effects. These bioactivities are believed to be attributed to various biologically active substances contained in snail mucus. Snail mucus comprises two types of polysaccharides, mucin and lectin, as well as seven types of glycoproteins, and other active components such as glycolic acid, allantoin, and antimicrobial peptides. In particular, the proteins present in snail mucus consist of 18 amino acids, among which eight are essential amino acids: isoleucine (Ile), leucine (Leu), lysine (Lys), methionine (Met), phenylalanine (Phe), threonine (Thr), valine (Val), and tryptophan (Trp). These components suggest the potential of snail mucus as a functional material for improving skin health, including regeneration, moisturization, and anti‐wrinkle effects (De Almeida et al. 2023; Li et al. 2024; Zhu et al. 2024). Clinical studies have demonstrated that topical application of snail secretion improves skin hydration, elasticity, and recovery after dermatological procedures. Nevertheless, evidence for the efficacy of oral administration of snail extracts remains scarce, highlighting the need for further investigation (Di Filippo et al. 2024; Theerawattanawit et al. 2021).

In the present study, we investigated the effects of orally administered edible snail extracts on skin hydration and the recovery of skin barrier function. Our central hypothesis is that oral snail extract powder (SEP) improves skin hydration by upregulating HA synthesis and barrier‐related genes. Our results showed that oral intake of SEP significantly boosted skin moisture levels and helped fix damaged skin barrier function in hairless SKH‐1 mice. We also assessed the safety of oral SEP consumption using the animal model.

## 2 Materials and Methods

### 2.1 Preparation of SEP Sample

The SEP powder was obtained from Age at labs (Chungcheong‐do, Republic of Korea). The edible SEP (Lot. number: A2) used in this study was derived from the edible snail *Achatina fulica* (Bowdich, Achatinidae), and was produced on July 27, 2024.

Raw material was washed and visually inspected to remove extraneous matter. After adding purified water, a first extraction was performed at ≥ 100°C. The extract was cooled to 50°C ± 2°C and subjected to enzymatic hydrolysis using food‐grade enzymes: proteases and α‐amylase. Reactions were carried out at pH 6.5 ± 0.5 for 10–12 h with agitation. The enzyme‐to‐substrate (E/S) ratio was controlled within the validated window of 0.4%–1.0% (proteases) and 0.04%–0.1% (α‐amylase) (w/w to raw material). Hydrolysis was terminated by thermal inactivation at 95°C–100°C for 10–15 min, followed by fine filtration (≥ 300‐mesh), concentration, and—when applicable—spray‐drying to yield a powder.

### 2.2 Lysine and Leucine Quantification by HPLC

Total lysine and leucine were determined after complete acid hydrolysis. Briefly, ~50 mg of sample (dry basis) was hydrolyzed in 6 M HCl containing 1% phenol at 110°C for 24 h under nitrogen. Hydrolysates were evaporated to dryness, neutralized, and reconstituted in borate buffer (pH 8.6) containing L‐norleucine as the internal standard. Amino acids were pre‐column derivatized with 6‐aminoquinolyl‐N‐hydroxysuccinimidyl carbamate (AQC; AccQ‐Tag chemistry) at 55°C for 10 min, then separated by RP‐HPLC (C18, 4.6 × 150 mm, ≈3–5 μm). Mobile phase A: aqueous buffer per manufacturer's protocol; mobile phase B: acetonitrile/water. A linear gradient at ~1.0 mL min^−1^ was applied; fluorescence detection was used (Ex 250 nm/Em 395 nm). Calibration was performed with certified lysine and leucine standards (≥ 6 levels), and results were expressed as mg g^−1^ (dry basis). For free lysine/leucine (optional), the same derivatization/HPLC conditions were used without prior acid hydrolysis. Method performance (linearity, precision, spike recovery) was verified before sample analysis.

### 2.3 Animal Experiments

**FIGURE 1:**
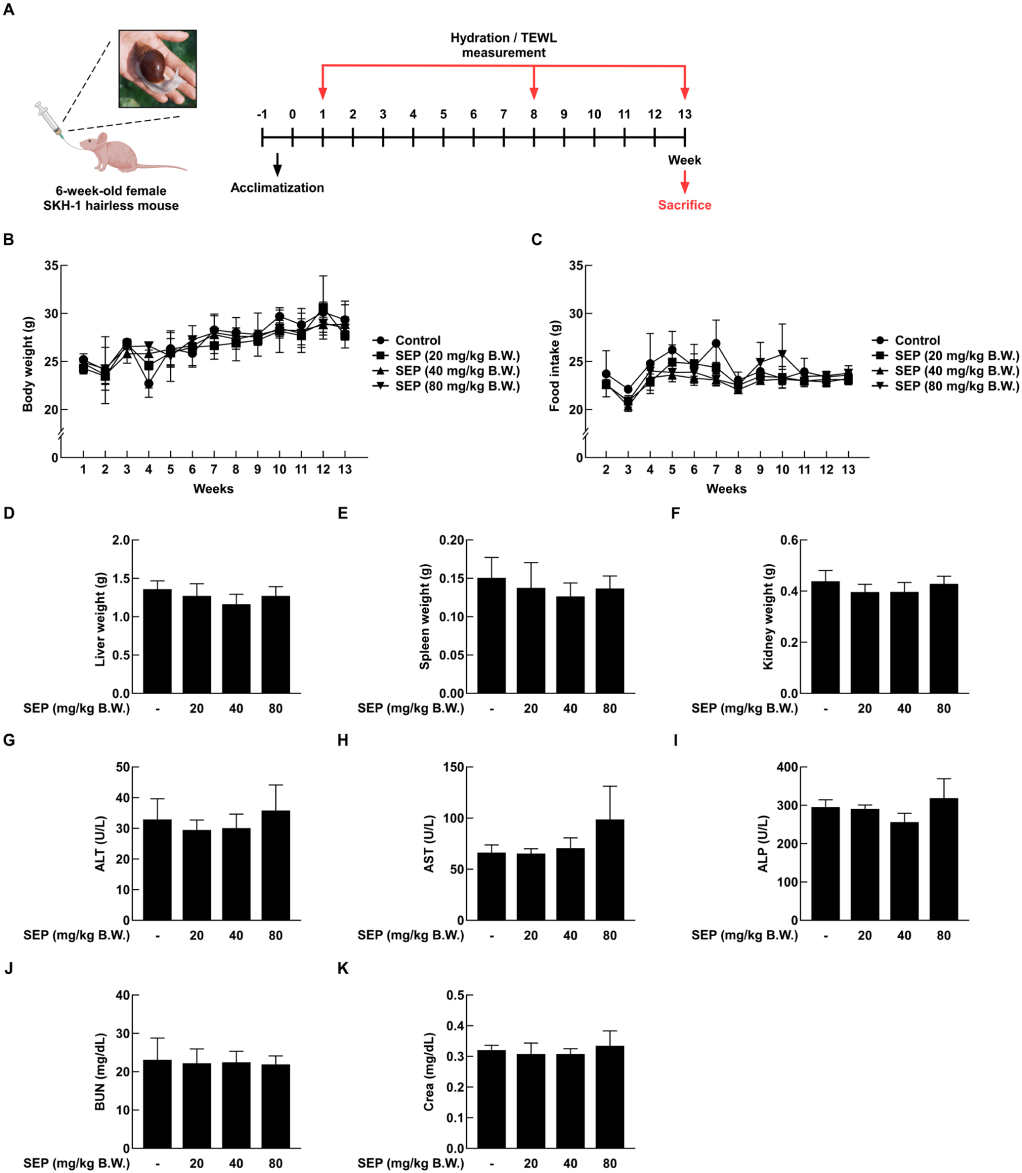
No significant toxicity was observed when SEP was administered orally to SKH‐1 null mice. (A) Schematic representation of the in vivo experimental design. (B, C) In this study, body weight change and feed intake were measured weekly for 13 weeks to evaluate the ingestive safety of SEP (*n* = 8). (D–K) After 13 weeks of treatment, mice were euthanized, and liver, spleen, and kidney weights were measured to assess organ toxicity. In addition, blood was collected from mice to determine liver toxicity and analyzed for liver toxicity biomarkers ALT, AST, and ALP. Blood urea nitrogen (BUN) and creatinine (Crea) concentrations were measured to assess renal function. All data were statistically analyzed by one‐way ANOVA followed by Dunnett's multiple comparison test.

Animal experiments were performed in accordance with the guidelines of the Institutional Animal Care and Use Committee of Sejong University, IACUC approval number SJ‐20241016‐01. Six‐week‐old female SKH‐1 mice were purchased from Orient Bio Inc. (Seongnam, Republic of Korea) and housed for 13 weeks in a controlled environment at 24°C ± 2°C, 55% ± 5% relative humidity, and a 12‐h light/dark cycle. After a week of acclimatization, the mice were randomly divided into four groups of eight. Several studies utilizing the SKH‐1 hairless mouse model for investigating skin hydration and photoaging have assessed the efficacy of oral or systemic interventions, wherein a group size of *n* = 8 per condition has been commonly employed as the standard experimental design (Son et al. 2020). The treatment groups received oral SEP by gavage at doses of 20, 40, and 80 mg/kg of body weight, while the control group was given saline by the same route. We performed oral administration once daily for a total of 13 weeks. Body weight and food intake were measured daily throughout the experimental period (Figure 1A). TEWL was checked with a TM300 probe (Courage and Khazaka GmbH, Cologne, Germany) at weeks 1, 8, and 13, and skin water content was measured using a Cutometer (Courage and Khazaka GmbH, Cologne, Germany).

### 2.4 Real‐Time Quantitative Polymerase Chain Reaction

Total RNA was extracted from dorsal skin tissue using TRIzol reagent (Thermo Fisher Scientific, Waltham, MA, USA). The extracted RNA was converted into cDNA using amfiRivert cDNA Synthesis Platinum Master Mix (GeneDEPOT, Katy, TX, USA). Real‐time quantitative PCR was performed under the following conditions: 95°C for 3 min of denaturation, followed by 95°C for 15 s of denaturation, 58°C for 15 s of annealing, and 72°C for 30 s of extension for a total of 40 cycles. The cycle threshold (Cq) of each gene was adjusted based on the Cq value of GAPDH, and the expression level of the target gene was compared to the control (1‐fold treatment). The primer sequences are shown in Table 1. Primer sequences for the target genes were designed according to their respective GenBank accession numbers. The specificity of each primer set was assessed using NCBI Primer‐BLAST, a widely utilized platform for primer design and specificity verification.

**TABLE 1:** Sequences of PCR‐primers.

Species	Gene symbols	Accession numbers	Forward (5′→3′)	Reverse (5′→3′)
*Mus musculus*	*Has1*	NM_008215.2	GTGCGAGTGTTGGATGAAGACC	CCACATTGAAGGCTACCCAGTATC
	*Has2*	NM_008216.3	GCCATTTTCCGAATCCAAACAGAC	CCTGCCACACTTATTGATGAGAACC
	*Has3*	NM_008217.4	GCTTCAGTCCAGAAACCAAAGTAGG	CCTCGTTCCTCAAGAGAAACAAGG
	*Hyal1*	NM_001331161.2	AAGTACCAAGGAATCATGCC	CTCAGGATAACTTGGATGGC
	*Col1a1*	NM_007742.4	GGGGCAAGACAGTCATCGAA	GAGGGAACCAGATTGGGGTG
	*Col3a1*	NM_009930.2	AAGGCTGCAAGATGGATGCT	GTGCTTACGTGGGACAGTCA
	*Tgfβ1*	NM_001289550.2	AGCACAGTATGCAAGCCTCG	ATCTGTAATGTTGAACTGGGTGG
	*Flg*	NM_001013804.2	CTAGAGGGCATGAGTGTAGTCA	CAAGACTGGACAGTTGGCTGG
	*Acer1*	NM_175731.4	TCTGAGGTGGATTGGTGTGAG	TGAGGGTCCAAAGATGAGGA
	*Cers2*	NM_001320492.1	ATGCTCCAGACCTTGTATGACT	CTGAGGCTTTGGCATAGACAC
	*Gapdh*	NM_001289726.1	CATCACTGCCACCCAGAAGACTG	ATGCCAGTGAGCTTCCCGTTCAG

### 2.5 HA ELISA Assay

HA content was measured using the Hyaluronan DuoSet ELISA kit (R&D Systems, Minneapolis, MN, USA) according to the manufacturer's protocol. Small pieces of skin tissue from the back were lysed using RIPA buffer, and the amount of protein was measured using the BCA (Bicinchoninic Acid) method. Equal amounts of protein and biotin‐labeled antibody were added at the same time to each well that had a capture antibody for HA, and then were allowed to react at 37°C for 45 min.

The plate was then washed three times with 1× washing solution, and HRP‐streptavidin conjugation solution was added to each well and incubated at 37°C for 30 min. After washing five times with washing solution, tetramethylbenzidine (TMB) substrate was added to each well, and the reaction was incubated for 15 min at 37°C in the dark. After adding the solution to stop the reaction, the absorbance was checked at 450 nm using a Cytation 1 instrument (BioTek, Winooski, VT, USA) at the Biopolymer Research Center for Advanced Materials.

### 2.6 Histological Analysis

Skin tissue analysis was outsourced to BioLead (Daegu, Korea). Dorsal skin tissues from mice were fixed in 4% paraformaldehyde, embedded in paraffin, and sectioned. The tissue sections were stained with 3,3′‐diaminobenzidine (DAB) to find out how much HAS2, HYAL1, and TGF‐β1 were present. The stained sections were examined with ImageJ software (NIH, Bethesda, MD, USA), and the amounts of HAS2, HYAL1, and TGF‐β1 were measured by comparing the stained area to the total tissue area.

### 2.7 Statistical Analysis

All experiments were performed at least three times, and representative results were presented as mean and standard deviation. Statistical significance was indicated using one‐way analysis of variance (ANOVA) with GraphPad Prism version 8.0.2 (GraphPad Software Inc., USA). Results were considered significant when the *p*‐value was less than 0.05.

## 3 Results

### 3.1 Oral Administration of SEP Does Not Induce Toxicity in SKH‐1 Hairless Mice

This study investigated the potential toxicity of orally administered SEP in SKH‐1 hairless mice. There were no significant differences in body weight between the control group and the SEP‐treated groups throughout the experimental period (Figure 1B). Similarly, food intake remained comparable between the groups (Figure 1C). Post‐sacrifice analysis of organ weights, including liver, spleen, and kidney, revealed no statistically significant differences between the control and treatment groups (Figure 1D–F). To assess potential systemic toxicity, serum levels of hepatic (ALT, AST, ALP) and renal (BUN, creatinine) biomarkers were measured (Figure 1G–K). No significant differences were observed between the control and SEP‐treated groups. These findings further support the in vivo safety of orally administered SEP.

### 3.2 SEP Enhances Skin Hydration

**FIGURE 2:**
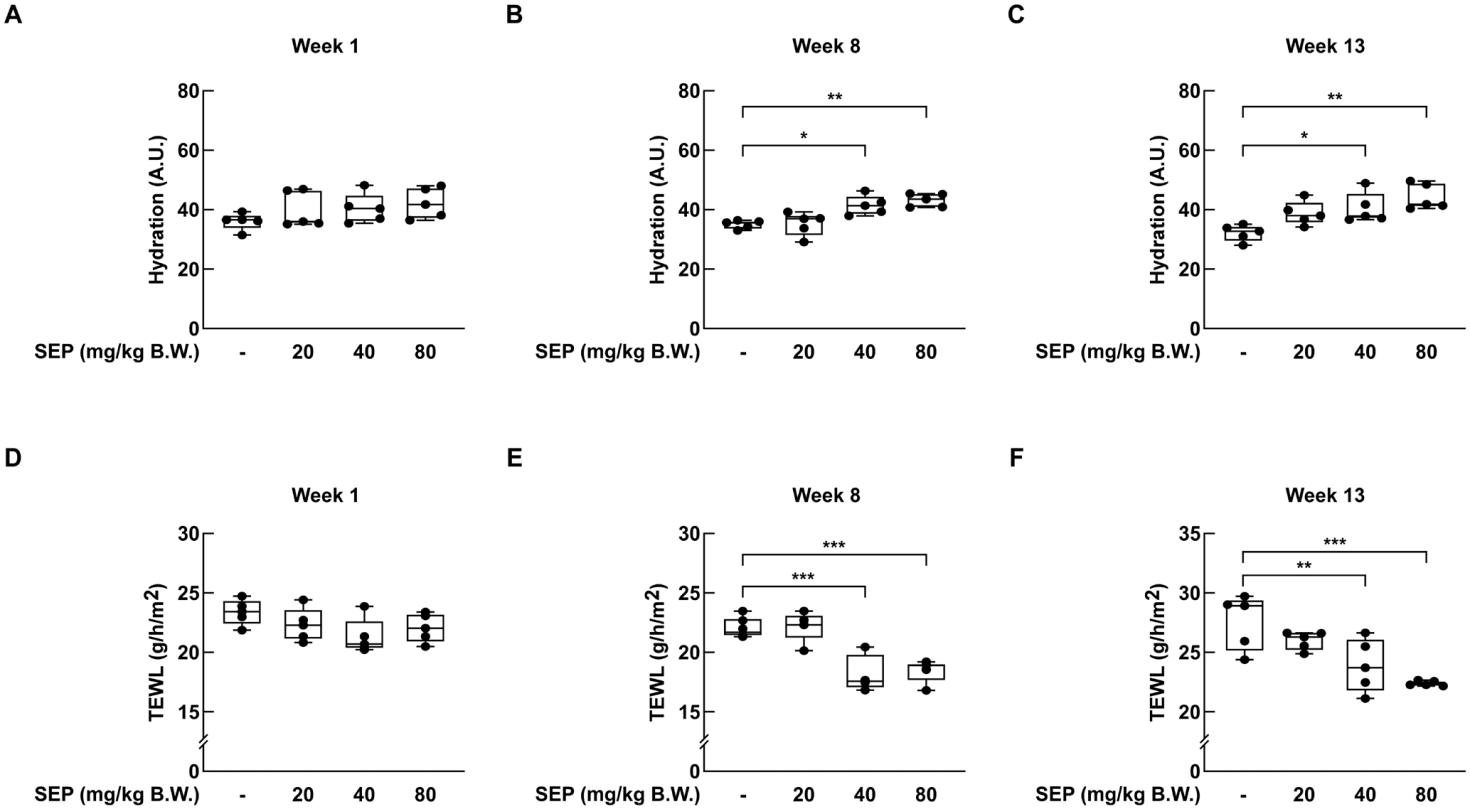
Oral administration of SEP significantly increased skin hydration in mice. Skin hydration levels following oral administration of SEP were assessed at the early (A), middle (B), and final (C) stages of the experiment using a Cutometer. Transepidermal water loss (TEWL) on the dorsal skin was measured at the early (D), middle (E), and final (F) stages using a TM300 probe. Statistical analysis was performed using one‐way ANOVA followed by Dunnett's multiple comparison test. The recovery effects of SEP on skin hydration and TEWL were evaluated in comparison to the non‐treated control group. Statistical significance was indicated as follows: *p < 0.05; **p < 0.01; ***p < 0.001.

To assess the effects of SEP on skin hydration, skin moisture parameters were measured at the early, mid, and late stages of the experiment. At the early stage, hydration levels were similar between the control and treatment groups (Figure 2A). However, during the mid and late stages, oral administration of SEP at 40 and 80 mg/kg B.W. led to a noticeable increase in skin hydration compared to the control group (Figure 2B,C). By the end of the study, the hydration level in the 80 mg/kg B.W. group reached 44.3 A.U., whereas the control group recorded 31.9 A.U.

A similar trend was observed in TEWL. While no differences were noted in the early phase (Figure 2D), TEWL values decreased significantly in the treatment groups during the mid and late phases (Figure 2E,F). At the endpoint, the 80 mg/kg B.W. group showed a TEWL of 22.6 g/h/m^2^ compared to 27.0 g/h/m^2^ in the control group, indicating improved skin barrier function.

### 3.3 SEP Modulates the Expression of Skin Hydration‐Related Genes

**FIGURE 3:**
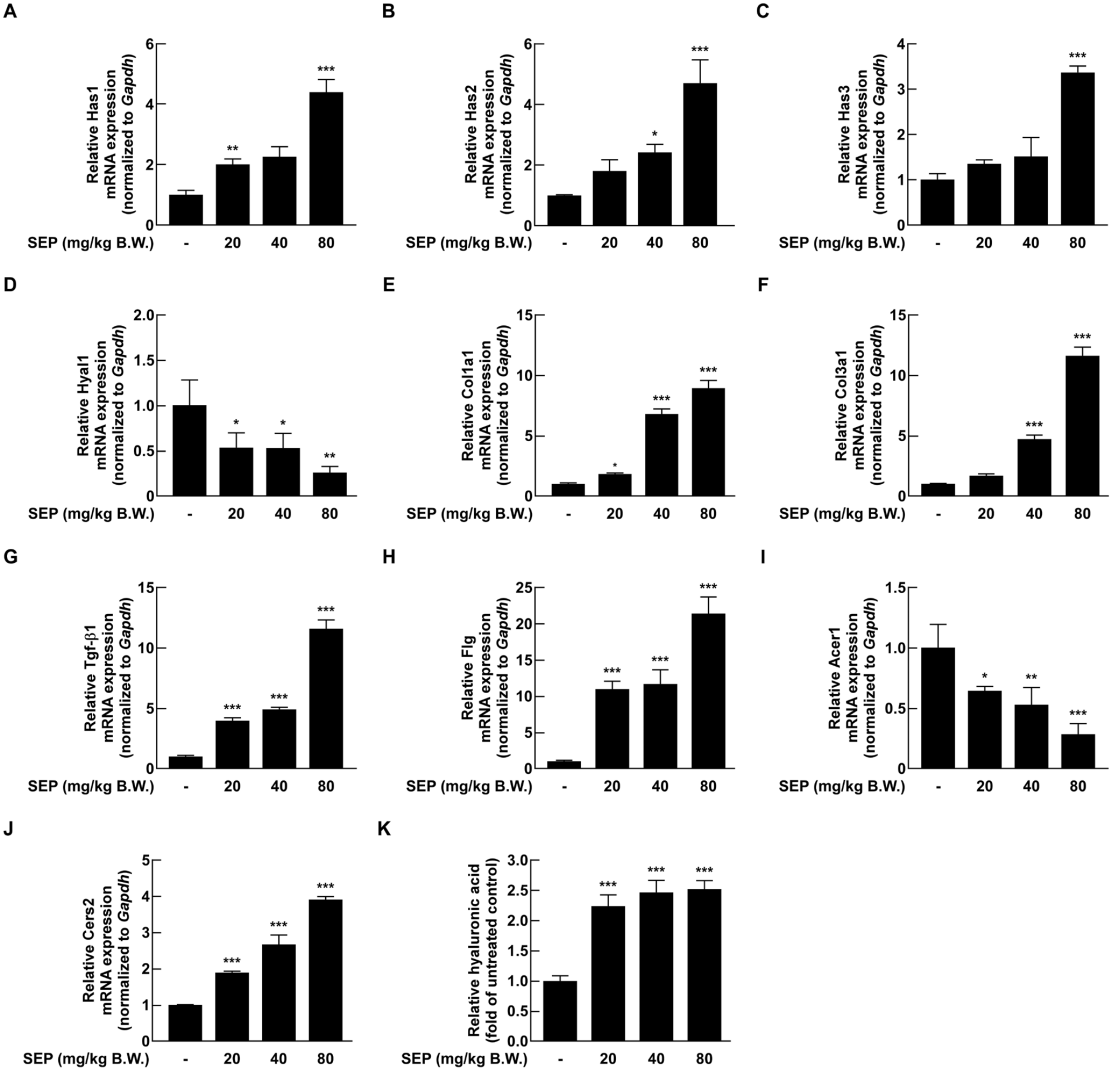
Oral administration of SEP modulated the expression of genes associated with skin hydration and skin barrier damage in mice. Total RNA was extracted from the dorsal skin tissues of mice using Trizol reagent, and the mRNA expression levels were analyzed by quantitative PCR. Genes related to hyaluronic acid metabolism, including *Has1* (A), *Has2* (B), *Has3* (C) (synthesis), and *Hyal1* (D) (degradation), were evaluated. Collagen‐related genes, *Col1a1* (E) and *Col3a1* (F), along with the regulatory and growth factor gene *Tgf‐β1* (G), were also analyzed. In addition, mRNA expression levels of skin barrier and moisturizing genes, *Flg* (H), *Acer1* (I), and *Cers2* (J) were quantified. Hyaluronic acid content in dorsal skin tissue lysates was measured using an ELISA assay (K). Statistical analysis was performed using one‐way ANOVA followed by Dunnett's multiple comparison test. Significant differences between the SEP‐treated and untreated groups were indicated as follows: *p < 0.05, **p < 0.01, ***p < 0.001.

To investigate the molecular mechanisms underlying the hydration‐enhancing effects of SEP, RT‐qPCR was performed on dorsal skin tissue. The mRNA expression of HA synthases, Has1, Has2, and Has3, was significantly upregulated in a dose‐dependent manner in the SEP‐treated groups (Figure 3A–C). On the other hand, the mRNA levels of Hyal1, which makes an enzyme that breaks down HA, were significantly lower when SEP was given (Figure 3D). In the same way, the genes that help make collagen, like Col1a1, Col3a1, and Tgf‐β1 mRNA, showed an increase in their activity depending on the amount given (Figure 3E–G). The mRNA expression of Flg, a key component of the skin barrier, was upregulated dose‐dependently (Figure 3H). The mRNA for ceramidase Acer1 was significantly reduced, while the mRNA for ceramide synthase Cers2 increased in the treatment groups (Figure 3I,J). These results suggest that SEP supports keeping the skin hydrated by regulating mRNA levels that are important for holding water, forming the extracellular matrix, and maintaining barrier function. To visualize the overall gene expression patterns, a heatmap was generated using all quantified mRNA expression values. This overview is provided in Figure S1.

### 3.4 SEP Increases HA Content in Mouse Skin

To further evaluate the effect of SEP on skin hydration, the HA content in mouse skin was quantified via ELISA. A dose‐dependent increase in HA levels was observed in the treatment groups compared to the control (Figure 3K). Specifically, the 20, 40, and 80 mg/kg B.W. groups exhibited HA concentrations of 121,646, 133,972, and 136,944 ng/mg, respectively, compared to 54,228.1 ng/mg in the control group. These values represent approximately 2.2‐, 2.4‐, and 2.5‐fold increases relative to the control. These findings align with the gene expression data, confirming that SEP enhances HA accumulation in the skin.

### 3.5 SEP Modulates HAS2, HYAL1, and TGF‐β1 Expression in Mouse Dorsal Skin

**FIGURE 4:**
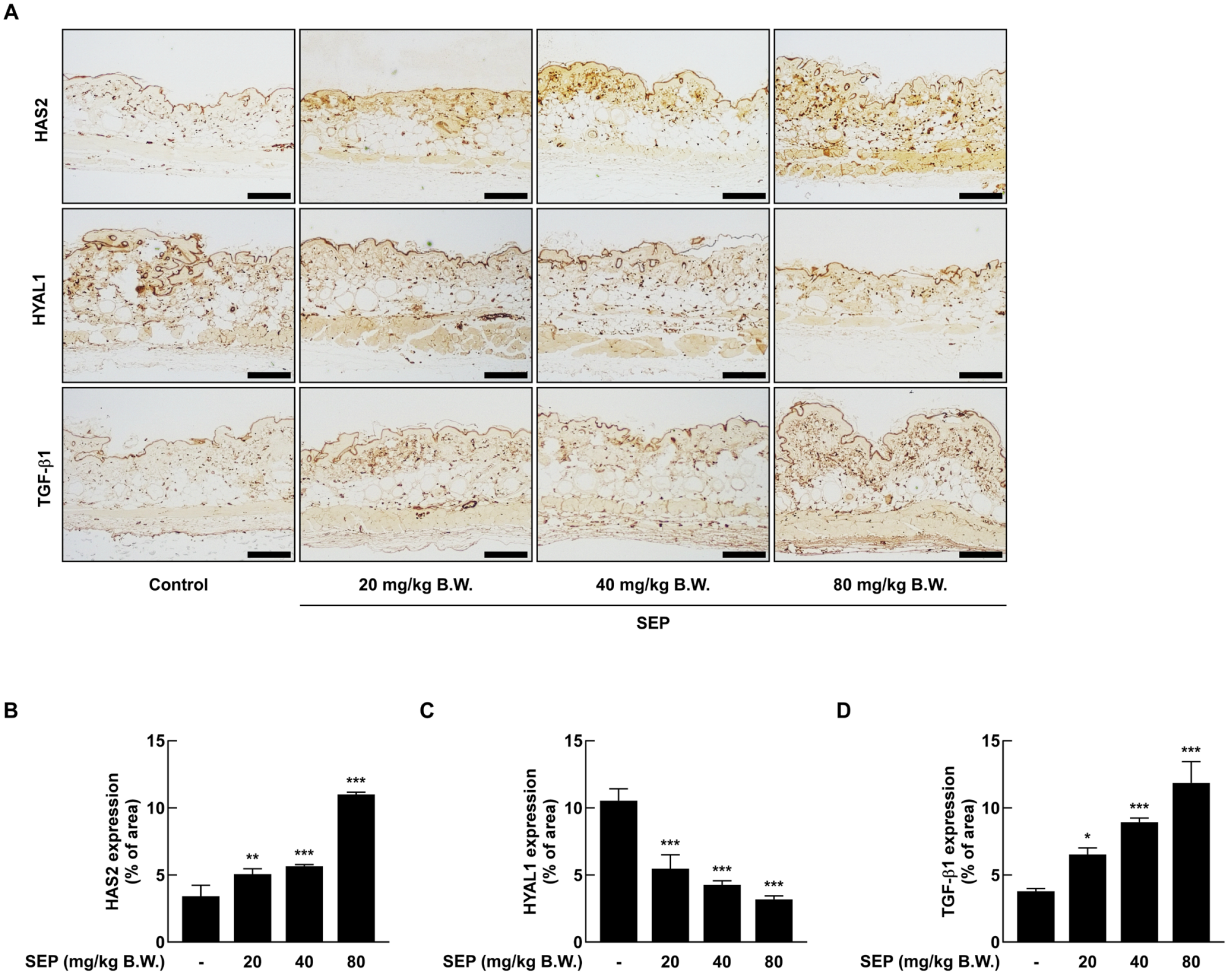
Oral administration of SEP was found to enhance skin elasticity and increase skin moisture content. The decreased expression of HYAL1 and the increased expression of HAS2 and TGF‐β1 in the dorsal skin tissues of mice were analyzed by immunohistochemistry (A). The intensity of protein expression in the skin tissues was quantitatively measured using ImageJ software (B–D). Statistical analysis was performed using one‐way ANOVA, followed by Dunnett's multiple comparison test. Significant differences between the SEP‐treated and untreated groups were indicated as follows: *p < 0.05, **p < 0.01, ***p < 0.001.

To assess the effects of SEP on skin hydration and extracellular matrix composition at the protein level, immunohistochemical (IHC) staining was conducted on dorsal skin tissue in mice (Figure 4A). The analysis showed that the levels of HAS2 protein increased significantly with more SEP treatment compared to the control group, while the levels of HYAL1 protein decreased steadily (Figure 4B,C). These findings match the results from RT‐qPCR and ELISA, confirming that SEP boosts the production of HA while reducing its degradation. Additionally, the amount of TGF‐β1 increased with higher doses in the treatment groups (Figure 4D), which matches the gene expression data. These findings suggest that oral administration of SEP promotes extracellular matrix remodeling.

## 4 Discussion

This study showed that giving oral edible SEP helps keep skin moist and strengthens its protective layer in SKH‐1 hairless mice. As a natural bioactive compound rich in glycoproteins and polysaccharides, SEP shows promise as a functional food ingredient for skin health (Zhu et al. 2024).

SEP greatly boosted skin moisture and reduced TEWL based on the amount used, especially at 80 mg/kg B.W., showing better skin moisture balance (Alexander et al. 2018; Chen et al. 2024). These improvements were mechanistically supported by upregulation of HA synthase genes (Has1, Has2, Has3), downregulation of hyaluronidase (Hyal1), and increased tissue levels of HA, as confirmed by ELISA. These findings reinforce the well‐established role of HA in water retention and epidermal barrier maintenance (Montero‐Vilchez et al. 2025). Also, SEP promoted the expression of genes related to collagen (Col1a1, Col3a1, Tgf‐β1) and skin barrier markers like Flg and Cers2, while suppressing Acer1 (Chen et al. 2024; Fu et al. 2024). These changes imply enhanced extracellular matrix integrity and barrier repair (Georgi et al. 2019; Lee et al. 2021; Melrose 2023). Immunohistochemical analysis showed that as the dose increased, there was more HAS2 and TGF‐β1 expression and less HYAL1, indicating changes at both the gene and protein levels. These results together suggest that SEP helps keep things moist by working together with processes related to hydration and the structure of tissues. The observed increase in collagen‐related gene expression may be attributable, in part, to the amino acid composition of SEP. Snail extract contains abundant glycine, proline, and lysine—key structural amino acids for collagen synthesis. Glycine contributes to the triple helix structure of collagen, while proline and hydroxyproline stabilize the helix, and lysine participates in collagen cross‐linking. These components may serve as substrates or cofactors that directly support collagen biosynthesis in vivo (de Paz‐Lugo et al. 2018; Salo and Myllyharju 2021).

While these findings are encouraging, it is important to acknowledge certain limitations. The SKH‐1 mouse model lacks fur and exhibits altered skin structure, which may not fully replicate the complexity of human skin physiology. Additionally, although barrier‐enhancing effects were clearly observed, the current study did not include inflammatory cytokine profiling (e.g., IL‐4, IL‐13, TNF‐α), and thus potential anti‐inflammatory contributions of SEP cannot be ruled out. Future studies should include cytokine and immune marker analysis to fully elucidate the immunomodulatory properties of SEP.

To evaluate translational relevance, we estimated the human equivalent dose (HED) based on FDA‐recommended Km conversion factors (Elmeliegy et al. 2021). The 80 mg/kg B.W. dose in mice corresponds to approximately 6.5 mg/kg in humans, or ~390 mg/day for a 60 kg adult. These estimates suggest that oral SEP may be developed as a nutraceutical candidate targeting dry skin or atopic dermatitis.

Importantly, no significant changes were observed in body weight, food intake, or major organ weights, suggesting that oral administration of SEP is safe over the tested duration. While topical application of snail mucin is widely used in cosmetic products, few studies have evaluated its systemic or oral efficacy. Our findings provide the first in vivo evidence that oral SEP supplementation improves skin hydration and barrier integrity through molecular and histological modulation, supporting its potential development as a skin health–focused functional food supplement.

## 5 Conclusion

This study demonstrates that oral administration of edible SEP significantly improves skin hydration and barrier function, primarily through upregulation of key genes such as HAS2 and FLG, and enhancement of HA and collagen synthesis. Among the tested doses, 80 mg/kg B.W. was identified as the optimal effective dose without toxicity. These findings suggest that SEP holds promise as an oral nutraceutical for managing xerosis and age‐related skin dryness, supporting its potential development as a functional food ingredient for skin health.

## Author Contributions

**Chaerin Lee:** investigation (equal), validation (equal), writing – original draft (equal). **Seoyoung Baek:** formal analysis (equal), writing – original draft (equal). **Wonchul Lim:** supervision (equal), writing – review and editing (equal). **Tae‐Gyu Lim:** writing – review and editing (equal), supervision (equal), conceptualization, funding acquisition.

## Conflicts of Interest

The authors declare no conflicts of interest.

## Supporting information


**Figure S1:** Heatmap visualization of hydration‐ and barrier‐related gene expression. Heatmap showing dose‐dependent changes in mRNA expression of Has1, Has2, Has3, Hyal1, Col1a1, Col3a1, Tgf‐β1, Flg, Acer1, and Cers2 in dorsal skin of SEP‐treated SKH‐1 mice. Gene expression was quantified by RT‐qPCR and normalized to control.

## Data Availability

The data that support the conclusions of this study are available upon request from the corresponding author.
