# Supplementary figures and images for: Orally Administered Edible Snail Extract Powder Enhances Skin Hydration via Hyaluronic Acid Synthesis and Barrier Gene Modulation in SKH‐1 Hairless Mice
Source: Food Sci Nutr. 2025 Oct 16;13(10):e71087. doi: 10.1002/fsn3.71087 (PMC12531416; doi:10.1002/fsn3.71087)

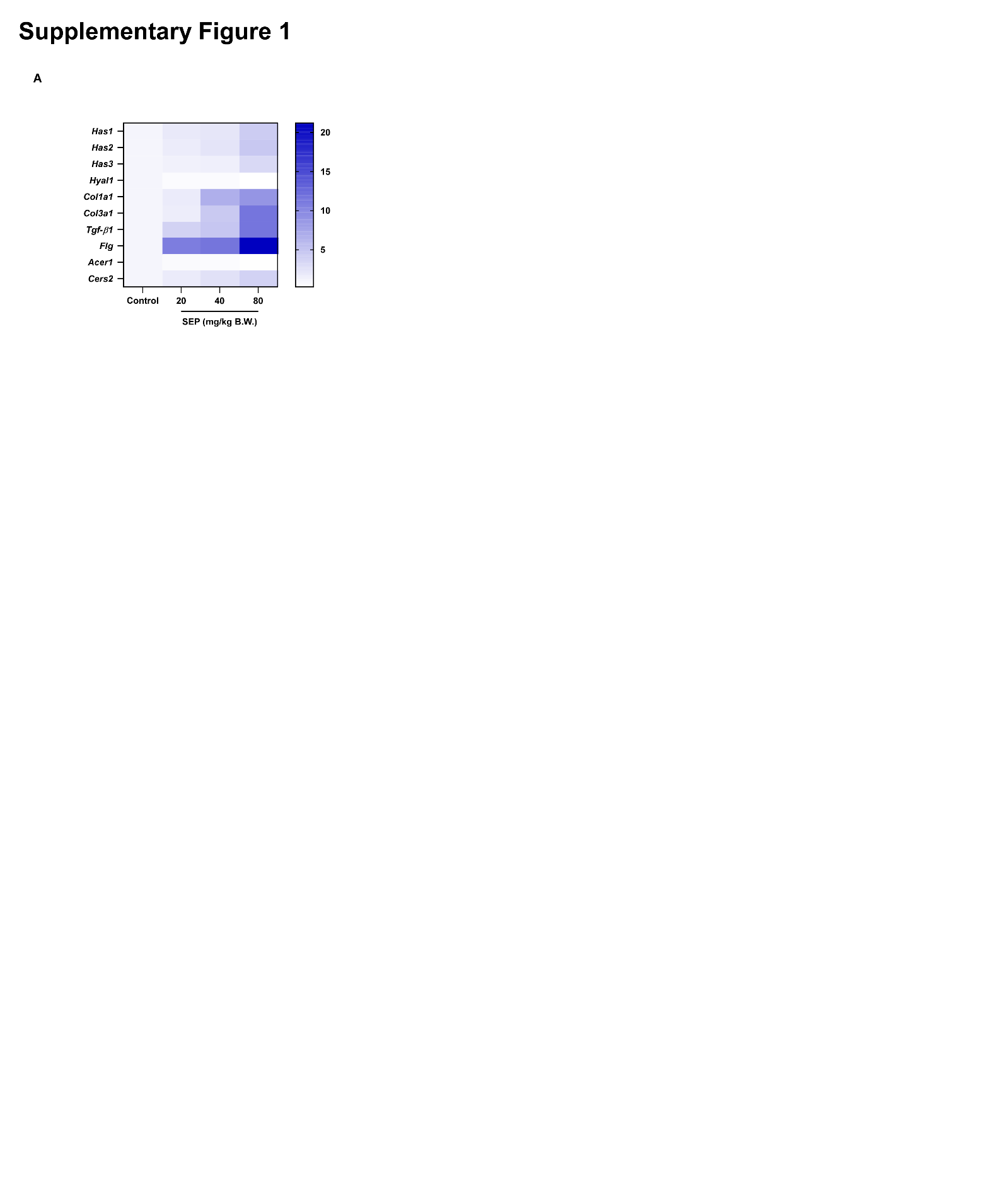

Supplement: Supplementary file 1 — Figure S1: Heatmap visualization of hydration‐ and barrier‐related gene expression. Heatmap showing dose‐dependent changes in mRNA expression of Has1, Has2, Has3, Hyal1, Col1a1, Col3a1, Tgf‐β1, Flg, Acer1, and Cers2 in dorsal skin of SEP‐treated SKH‐1 mice. Gene expression was quantified by RT‐qPCR and normalized to control. [file FSN3-13-e71087-s001.docx]
